# Optimized In Silico Modeling of Drug Absorption after Gastric Bypass: The Case of Metformin

**DOI:** 10.3390/pharmaceutics13111873

**Published:** 2021-11-05

**Authors:** Arik Dahan, Daniel Porat, Milica Markovic, Moran Zur, Olga Kister, Peter Langguth

**Affiliations:** 1 Department of Clinical Pharmacology, School of Pharmacy, Faculty of Health Sciences, Ben-Gurion University of the Negev, Beer-Sheva 8410501, Israel; poratdan@post.bgu.ac.il (D.P.); mmarkovic@unmc.edu (M.M.); moranfa@post.bgu.ac.il (M.Z.); 2 Department of Biopharmaceutics and Pharmaceutical Technology, Johannes Gutenberg University Mainz, 55099 Mainz, Germany; olga.kister@web.de

**Keywords:** bariatric surgery, Roux-en-Y gastric bypass, obesity, metformin, GastroPlus^TM^, intestinal permeability, segmental-dependent absorption, program simulation

## Abstract

Bariatric surgery is an effective treatment for severe obesity and related comorbidities, such as type II diabetes. Gastric bypass surgery shortens the length of the intestine, possibly leading to altered drug absorption. Metformin, a first-line treatment for type II diabetes, has permeability-dependent drug absorption, which may be sensitive to intestinal anatomic changes during bypass surgery, including Roux-en-Y gastric bypass (RYGB). Previous computer simulation data indicate increased metformin absorption after RYGB. In this study, we experimentally determined the region-dependent permeability of metformin, using the rat single-pass intestinal perfusion method (SPIP), which we then implemented into GastroPlus^TM^ to assess the contribution of our SPIP data to post-RYGB metformin absorption modeling. Previous simulations allowed a good fit with in vivo literature data on healthy and obese control subjects. However, it was revealed that for post-RYGB drug absorption predictions, simply excluding the duodenum/jejunum is insufficient, as the software underestimates the observed plasma concentrations post-RYGB. By implementing experimentally determined segmental-dependent permeabilities for metformin in the remaining segments post-surgery, GastroPlus^TM^ proved to fit the observed plasma concentration profile, making it a useful tool for predicting drug absorption after gastric bypass. Reliable evaluation of the parameters dictating drug absorption is required for the accurate prediction of overall absorption after bariatric surgery.

## 1. Introduction

Since the 1980s the prevalence of obesity has more than doubled, and is now a global epidemic. Obesity is related to many comorbidities, including type 2 diabetes, hypertension, dyslipidemia, and sleep apnea [1], as well as unfortunate outcomes that lead to a shorter life expectancy [2]. Since diet and exercise alone cannot quite achieve the desired weight loss, there is a need for other, long-term, effective treatments for obesity.

**Figure 1:**
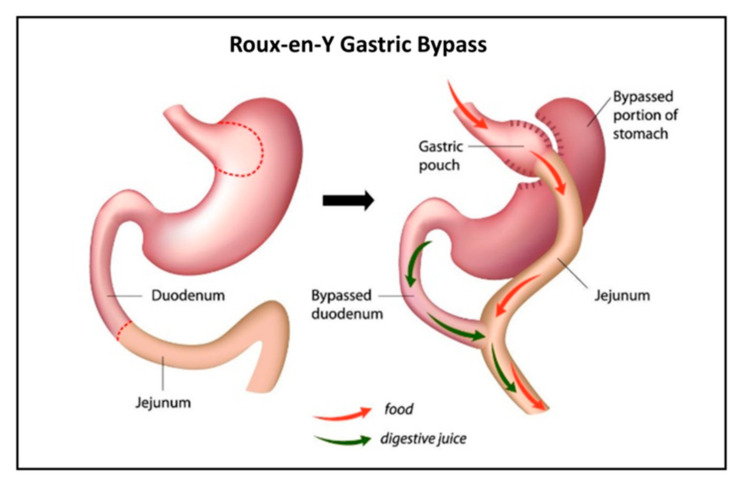
Illustration of Roux-en-Y gastric bypass (RYGB).

For patients with severe obesity (BMI > 40), there are currently few therapies or pharmaceuticals offering lasting weight loss. In these cases, bariatric surgery is suggested [3]. There are several available bariatric surgical techniques, with Roux-en-Y gastric bypass (RYGB) long being the gold standard [4]. The operation results in a smaller gastric pouch to restrict oral intake and the construction of an intestinal limb where bile and pancreatic fluid are diverted from the proximal to distal intestine (Figure 1) to limit food absorption. Meanwhile, the digestion and absorption of nutrients change as well, often leading to nutritional deficiencies [5]. With this in mind, there is ample reason to suspect similar undesired effects of RYGB on the absorption of drugs [6,7]. Indeed, data from the literature have been emerging in recent years regarding drug disposition following bariatric surgery [8]. Yet, only few drugs were studied and compared for their pre- vs. post-surgery absorption [9].

One of these drugs is metformin, a first-line treatment for type 2 diabetes, a common comorbidity of obesity. Padwal et al. have reported an increased bioavailability of metformin after RYGB (*n* = 16 in each group) [10]. This was in contrary to their hypothesis, which stated that the amount of metformin absorbed would be significantly smaller after the surgery since the duodenum and proximal jejunum are bypassed. To explain their results they suggested additional mechanisms, including increased transit time, transporter upregulation, and intestinal adaptation resulting from villous hyperplasia [10].

**Figure 2:**
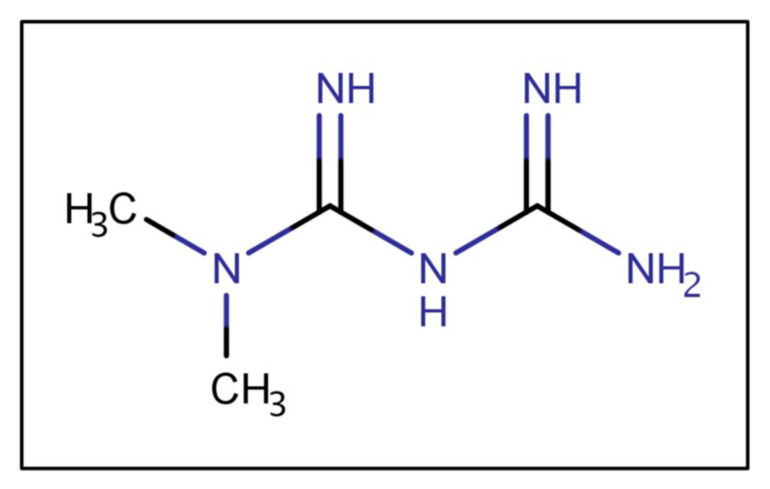
Metformin’s chemical structure.

Metformin is a biguanide (Figure 2), a strong base, and in typical gastric pHs is protonated, bearing a positive charge. As a cationic, hydrophilic drug, metformin is a substrate of various intestinal organic cation transporters [11]. The ionized metformin has a tendency to stick to the intestinal wall since the epithelium is negatively charged [12]. Data suggest that high concentrations of metformin are retained in the upper parts of the GI tract for several hours, leading to depot-like behavior [13,14,15]. The accumulation of metformin within the intestinal wall could reduce the concentration gradient governing passive absorption, overall decreasing bioavailability [16]. The low absorption rate from the duodenum, jejunum, and ileum could maintain high metformin concentrations in the small intestine [17]. The intestinal absorption is site-dependent and decreases along the intestine (duodenum > jejunum > ileum) [18]. Moreover, metformin has poor colonic absorption [12].

The purpose of this study was to reinvestigate the reported increased bioavailability of metformin after RYGB [10] using GastroPlus^TM^, and to show a novel approach that predicts the unexpectedly increased metformin bioavailability previously reported [10].

## 2. Materials and Methods

### 2.1. Materials

Metformin, potassium phosphate monobasic, and sodium phosphate dibasic were purchased from Sigma Chemical Co. (St. Louis, MO, USA). Water and acetonitrile (Merck KGaA, Darmstadt, Germany) were ultraperformance liquid chromatography (UPLC) grade, as was trifluoroacetic acid (TFA), purchased from Sigma Chemical Co. All other chemicals were of analytical reagent grade.

### 2.2. Rat Single-Pass Intestinal Perfusion (SPIP)

The single-pass intestinal perfusion (SPIP) method was used to determine the rat effective permeability coefficient (*P_eff_*) of metformin vs. metoprolol in different intestinal regions [19,20]. Animal studies were performed using protocols approved by the Ben-Gurion University of the Negev Animal Use and Care Committee (Protocol IL-07-01-2015). Male Wistar rats (weighing 230–260 g, Harlan, Israel) were housed and handled according to Ben-Gurion University of the Negev Unit for Laboratory Animal Medicine Guidelines. The study protocol used for animal experimentations followed previous reports [21,22,23,24]. In brief, anesthetized rats were placed on a 37 °C surface (Harvard Apparatus Inc., Holliston, MA, USA), and a 3 cm midline abdominal incision was made. Due to the unique luminal conditions of each intestinal segment, the metformin permeability through three different segments (length of 10 cm each) was measured: jejunum (starting 2 cm below the ligament of Treitz), middle small intestinal segment (mid SI), and ileum [25,26]. Each segment was cannulated on both sides and perfused with the relevant blank buffer (freshly prepared 30 min prior to starting the experiments by adding different ratios of potassium phosphate monobasic and sodium phosphate dibasic to obtain desired pH values). Osmolality (290 mOsm/L) and ionic strength (50 mM) were maintained in a similar fashion in all buffers. Phosphate buffers containing metformin and metoprolol were prepared at a pH of 6.5, 7.0, and 7.5, followed by incubation in a 37 °C water bath. The pH of each solution matched the physiological pH of the intestinal segment studied (jejunum, pH of 6.5; mid SI, pH of 7.0; and ileum, pH of 7.5). The drug-containing buffer (50 μM) was perfused through the intestinal segment (Watson Marlow 205S, Watson-Marlow Bredel Inc., Wilmington, MA, USA) at a flow rate of 0.2 mL/min for 1 h to ensure steady-state conditions; the perfusion continued for an additional 1 h, with samples taken every 10 min. The pH of the collected samples was measured at the outlet to verify that there was no pH change throughout the perfusion. All samples were immediately assayed by UPLC. The length of each perfused intestinal segment was measured at the experiment endpoint.

The effective permeability (*P_eff_*; cm/s) through the intestinal wall was calculated using Equation (1):

(1) $$P_{eff}=\frac{-Qln\;(C\prime_{out}/C\prime_{in})}{2\pi RL}\;$$

where *Q* is the perfusion buffer flow rate (0.2 mL/min), *C^′^_out_/C^′^_in_* is the ratio of the outlet and the inlet concentrations of drug, adjusted for water transport by the gravimetric method [27,28,29,30], *R* is the radius of the intestinal segment (set to 0.2 cm), and *L* is the length of the perfused segment.

### 2.3. Analytical Methods

Ultra-performance liquid chromatography (UPLC) was performed on a Waters (Milford, MA, USA) Acquity UPLC H-Class system equipped with a photodiode array detector (PDA) and Empower software. The determination of the investigated drugs and the non-absorbable marker, phenol red, in the SPIP samples was achieved using a Waters (Milford, MA, USA) Acquity UPLC BEH C18 1.7 μm 2.1 × 100 mm column. A gradient mobile phase consisted of 90:10 shifting to 20:80 (*v*/*v*) water:acetonitrile (both with 0.1% TFA) over 7 min. The detection wavelengths for metformin and metoprolol were 229 and 275 nm, respectively. Injection volumes ranged from 2 to 50 μL. All the analytical methods were adequately validated in the range of experimental concentrations and complied with the accepted standards of accuracy, precision, and linearity.

### 2.4. Gastrointestinal Simulations

GastroPlus^TM^ software (version 9.5 Simulations Plus, Inc., Lancaster, CA, USA) was used for gastrointestinal simulations of metformin absorption in healthy subjects, as well as subjects with obesity. GastroPlus^TM^ is a mechanistically based simulation software that can predict absorption, pharmacokinetics, and pharmacodynamics in human/animal models. It is based on the Advanced Compartmental Absorption and Transit (ACAT) model that consists of nine intestinal compartments, and accounts for all relevant parameters that may impact oral drug absorption (physicochemical drug properties, formulation design, physiological conditions, and drug pharmacokinetic data) [31]. The simulations were performed on an HP Laptop with an Intel Core i3 (2.4 GHz). The ADMET Predictor (V. 8.1, Simulations Plus, Inc., Lancaster, CA, USA) predicted the physiological parameters of metformin using metformin’s chemical structure (Figure 2). Additional parameters were taken from the literature. The constant parameters for each simulation are presented in Table 1. It is known that metformin is predominantly transported via the paracellular route (approximately 90%) [32,33]. Therefore, the paracellular permeability was included into GastroPlus^TM^ simulations. The used paracellular model followed previously published reports [34]. In order to adjust the ratio between the paracellular/transcellular *P_eff_* to 90%:10%, the molecular radius was altered.

### 2.5. Metformin Simulations

#### 2.5.1. Metformin in the Healthy Subjects

Initially, in order to validate the produced model, we performed a simulation of metformin’s PK profile, based on a study by Pentikäinen et al. [35]. In that study, an oral dose of metformin (500 mg tablet) taken with 200 mL of water was given to a 63.4 kg patient (a mean value of 5 subjects was used in the input simulation parameters). Based on the selected set of input data (Table 1), we produced a suitable model predicting the plasma concentration–time profile of metformin.

#### 2.5.2. Metformin Control Subjects (Individuals with Obesity)

Padwal et al. reported metformin absorption for individuals with obesity (BMI = 40.5) and post-RYGB patients [10]. Individuals with obesity were used as a control group for the simulations, while dose, body weight, renal clearance, and the volume of distribution were adjusted, according to Table 1.

#### 2.5.3. Development of a Post-RYGB Physiology Model

To simulate the physiology after RYGB in GastroPlus^TM^, a post-RYGB physiology in the gut physiology tab of GastroPlus^TM^ was created. The upper small intestinal compartments (duodenum and jejunum 1) were omitted by setting the intestinal transit time, volume, and length to zero, thereby mimicking the conditions following gastric bypass. The altered physiology parameters can be found in Table 2. The transit time and stomach volume were decreased since RYGB surgery involves the creation of a smaller gastric pouch. Following the operation, acid secretion from the stomach is decreased [42], resulting in higher gastric pH [43]. Therefore, the pH was increased (Table 2). In addition, the dose, body weight, renal clearance, and volume of distribution for the RYGB group were adjusted (Table 1).

#### 2.5.4. Development of an Adjusted Physiology following RYGB

Since the developed model did not demonstrate suitable fitting to the observed plasma concentration, further adjustments had to be made. The first step was to include the measured segmental-dependent rat metformin permeability values into the post-RYGB physiology. The duodenal permeability for the simulations was taken from Song et al. [18]. In the compound tab of GastroPlus^TM^ the effective permeability can be entered in different ways; since part of the jejunum is missing in the post-RYGB subject, it is important to include the permeability values for the other segments of the GIT. The permeability data were measured in rats (*P_eff,rat_*); therefore, the human intestinal permeability (*P_eff,man_*) was predicted according to Equation (2) [44]:

(2) $$P_{eff,man}=3.6\times P_{eff,rat}+0.03\times 10^{-4}\;$$

All other parameters were held constant. Data for each segment were entered into the gut physiology tab in GastroPlus^TM^.

## 3. Results

### 3.1. Rat Intestinal Perfusion Studies

**Figure 3:**
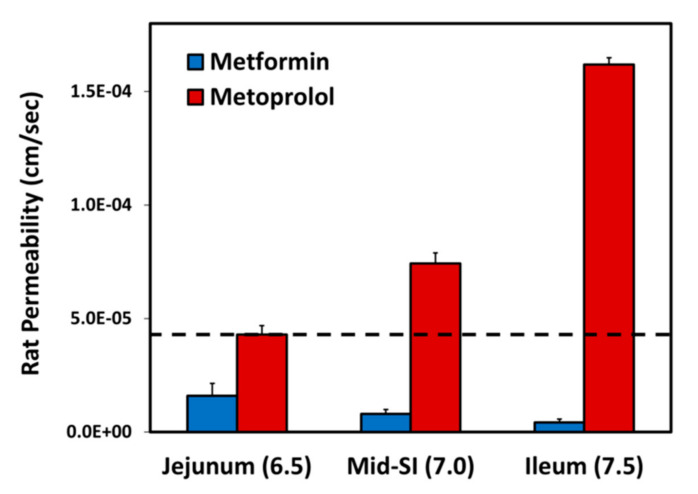
Effective permeability coefficient (*P_eff_*; cm/s) obtained for metformin vs. metoprolol in three rat intestinal segments: jejunum (pH of 6.5), mid small intestine (SI) (pH of 7.0), and ileum (pH of 7.5). The jejunal permeability of metoprolol is a low/high-permeability class boundary and is illustrated by the black dashed line. Data are presented as means ± S.D; *n* = 6.

The effective permeability coefficient (*P_eff_*) values for metformin vs. metoprolol determined using the single-pass rat intestinal perfusion model are presented in Figure 3. Permeability studies were performed in three intestinal segments with their corresponding pH: the jejunum (pH of 6.5), the mid small intestine (SI) (pH of 7.0), and the ileum (pH of 7.5). Metformin exhibits downward segmental-dependent permeability throughout the lumen of the SI, as the permeability of metformin in the jejunum was higher than that in the ileum. The permeability of metoprolol in the jejunum (pH of 6.5) is considered a low/high-permeability class boundary marker (marked as a dashed line in Figure 3) [45]; at any given intestinal segment/pH, the permeability of metformin was lower than that of metoprolol in the jejunum (pH of 6.5), demonstrating that metformin is a low-permeability compound.

### 3.2. Simulations in a Healthy Subject

**Figure 4:**
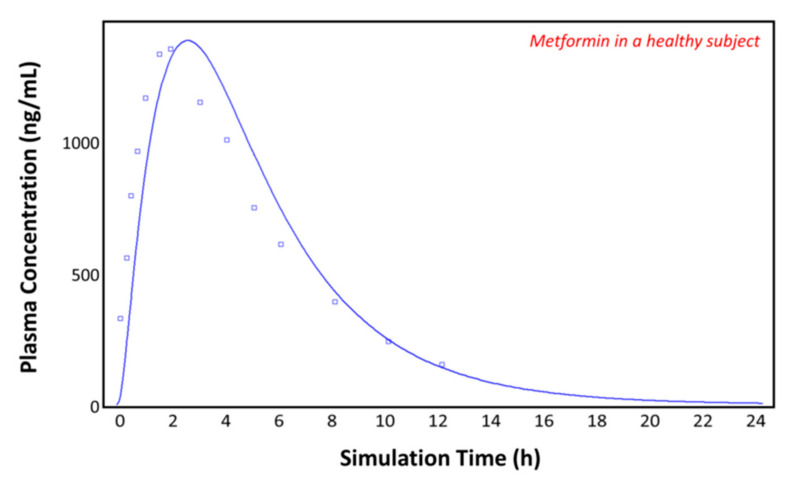
Plasma concentration–time profile observed by Pentikäinen et al. (squares) and the predicted plasma concentration–time profile by GastroPlus^TM^ (solid line) in a healthy subject.

**Figure 5:**
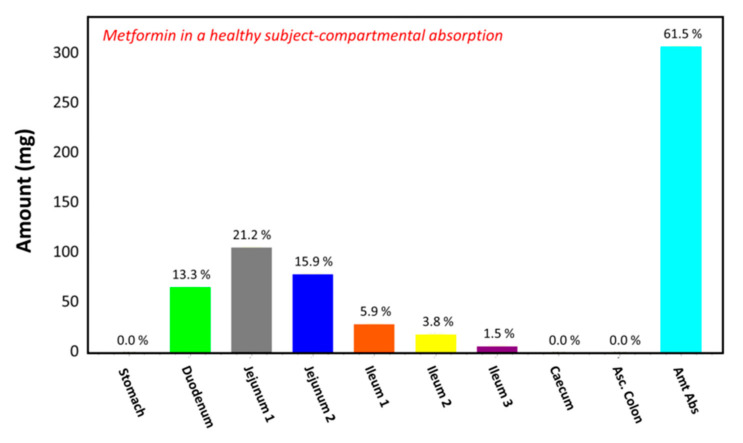
Regional gastrointestinal absorption predicted by GastroPlus^TM^ in healthy human subjects following oral administration of 500 mg of metformin.

The observed plasma concentration–time profile of healthy human subjects obtained by Pentikäinen et al. [35] (squares) and the plasma concentration–time profile predicted by GastroPlus^TM^ following oral administration of 500 mg of metformin (solid line) are presented in Figure 4. The simulation was performed using the single simulation mode and Human-Physiological-Fasted ACAT model. This model gave a good prediction of the plasma concentration–time profile of metformin in a healthy human subject. The extent of absorption in the intestinal compartments of the ACAT model is presented in Figure 5. The majority of the metformin dose is absorbed in the jejunum and the duodenum with very little absorption in the distal intestinal segments. This might indicate that the absorption of metformin following an RYGB procedure will be low, since the duodenum and the jejunum are bypassed.

### 3.3. Simulations in the Control (Individuals with Obesity) Subjects

**Figure 6:**
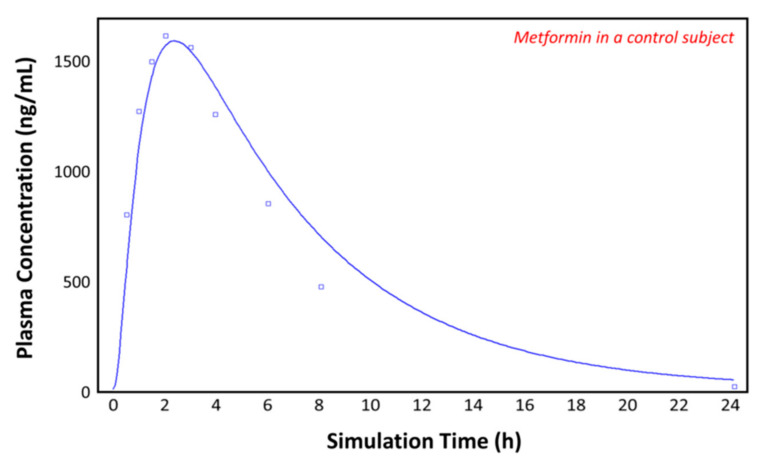
Plasma concentration–time profile observed by Padwal et al. (squares) and the predicted plasma concentration–time profile by GastroPlus^TM^ (solid line) in the control subject (with obesity).

The observed plasma concentration–time profile by Padwal et al. [10] (squares) for patients with obesity and the plasma concentration–time profile following oral administration of 1000 mg of metformin predicted by GastroPlus^TM^ (solid line) are presented in Figure 6. The simulation was performed using the single simulation mode and as the ACAT model Human-Physiological-Fasted. The model gives a good prediction of the plasma concentration–time profile of metformin in the control (individual with obesity) subject.

### 3.4. Simulations in the Post-RYGB Group

**Figure 7:**
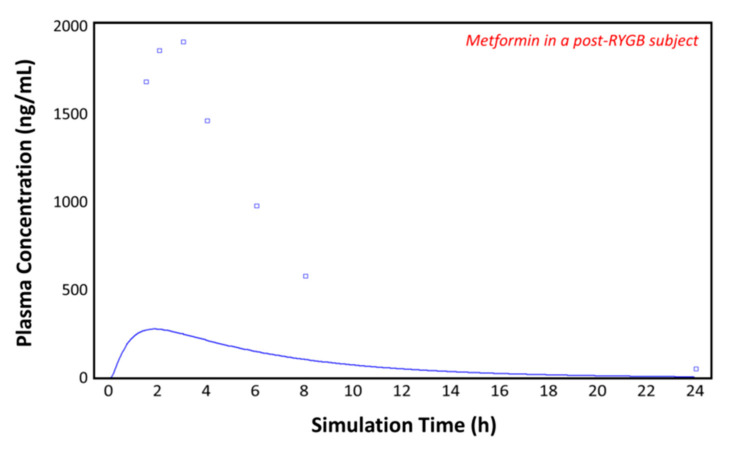
Plasma concentration–time profile observed by Padwal et al. (squares) and the predicted plasma concentration–time profile by GastroPlus^TM^ (solid line) in the post-RYGB subject.

Using the post-RYGB physiology as the ACAT model, the absorption of metformin was simulated with the parameters taken from Table 1 and Table 2. Figure 7 shows the plasma concentration–time profile measured by Padwal et al. 17 months (mean value) after RYGB surgery (squares) [10]. The predicted pharmacokinetic profile (solid line) clearly underestimates the observed metformin plasma concentrations (squares), suggesting that adaptations are made in the operated GI tract or that other parameters are involved. To fit the observed profile, further changes to the model were made.

### 3.5. Simulations in the Post-RYGB Group–Fitted

**Figure 8:**
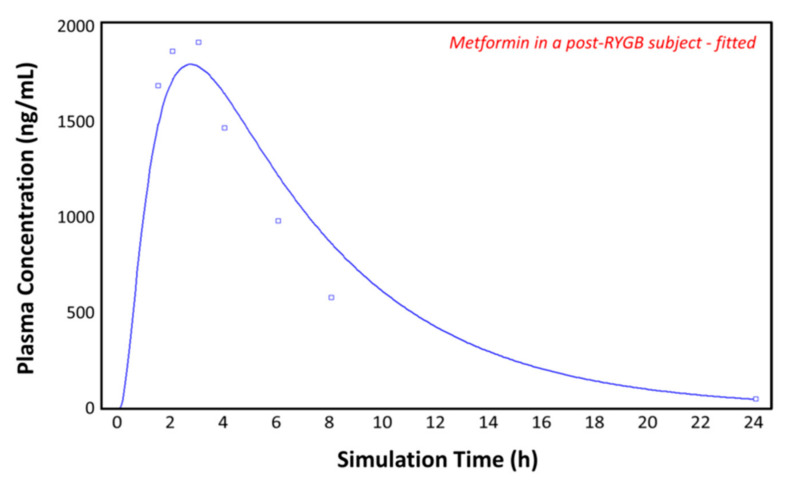
Plasma concentration–time profile observed by Padwal et al. (squares) and the predicted plasma concentration–time profile by GastroPlus^TM^ (solid line) in the post-RYGB subject with adjusted physiology.

Figure 8 again shows the plasma concentration–time profile measured by Padwal et al. 17 months (mean value) after RYGB (squares) [10]. By adding the segmental-dependent permeability values in the gut physiology tab for the remaining segments, GastroPlus^TM^ gives a good prediction of the observed plasma concentration–time profile. It appears that the permeability data allow good predications of metformin pharmacokinetics after RYGB, without having to change the pore size, porosity, absorptive surface area, or other parameters. The predicted pharmacokinetic parameters show a close fit to both the control and the post-RYGB group (Table 3).

## 4. Discussion

Since bariatric surgery is a major treatment for class 3 obesity patients with type 2 diabetes, it is vital to improve the treatment decision process after surgery to ensure better patient care and clinical outcomes [46,47]. Obesity is associated with many comorbidities, and these patients may be receiving multiple medications. Since the disease burden does not immediately vanish after surgery, patients often have to continue with their drug treatment (note: in many cases, anti-diabetic treatment is stopped after surgery) [48]. As the anatomy of the GI tract is changed, altered absorption may occur for different drugs. Despite that, there are only a few in vivo studies that investigated oral drug absorption following bariatric surgery.

One study found that, in contrast to their early prediction, the absorption of metformin was actually increased after surgery [10]. To explain this finding, they suggested four possible mechanisms: (1) RYGB increases intestinal transit time, thus prolonging the time of metformin residence in the small intestine [49]. Metformin is mainly absorbed from the small intestine, and its absorption is permeability rate-limited. Therefore, extending the duration of metformin exposure to the intestinal mucosa may increase overall absorption [37]. (2) The decreased acid secretion and therefore the increased pH in the newly formed gastric pouch should not have an effect on metformin solubility/dissolution, as it is a strong base; it may have effects on other, less basic drugs, whose pKa is lower and are poorly soluble. (3) Another possible mechanism is the upregulation of transporters. Metformin is a substrate for organic cation transporters (OCTs) found in the kidney and liver, as well as the plasma membrane monoamine transporter (PMAT) [50,51]. PMAT is found in the intestine and is pH sensitive. Only 10% of metformin is absorbed transcellularly, which is regulated by transporters, and 90% is absorbed paracellularly [33]. Since the absorption by the PMAT accounts for only a small percentage of metformin absorption and the upregulation of transporters by increasing the transporter expression in GastroPlus^TM^ showed no effect [34], we chose to exclude transporters from our model to focus on other possible reasons for the higher absorption after RYGB. (4) The last possible explanation was the small intestinal adaptation from villous hyperplasia. Various studies show that the intestine can adapt to the new anatomy after bypass surgery by increasing the intestinal epithelial surface area, involving increased functional capacity [52,53]. After RYGB surgery in rats, an increase in villus height and crypt cell proliferation adaptation is shown [54]. These adaptations seem to be the result of hormonal stimuli [55,56]. Another study concludes that for a subgroup of patients, intestinal permeability significantly increases after RYGB [57].

Almukainzi et al. analyzed the results of Padwal et al. using the simulation software GastroPlus^TM^ [34]. They created simulated post-surgery physiological conditions to assess which parameters may have caused the findings of Padwal et al. Their study suggests that the bioavailability of metformin is increased as a result of adaptations by the body, expressed as enlarged pore size, porosity, and the absorptive area in the remaining parts of the intestine [34]. Their simulation shows a good fit to the observed plasma concentration [10], while other data suggest that the adaptation might not be the only reason for enhanced metformin exposure [12]. Unlike our compartmental model, Almukainzi et al. created a PBPK model based on age and body weight, with permeability-limited tissues in addition to the inclusion of transporters such as OCTs and the PMAT [34]. Since we knew the body weight, the renal clearance, and the volume of distribution for the observed plasma concentration–time profiles before and after RYGB surgery [10], we could recreate a simulation using only the compartmental model, focusing on the segmental-dependent permeabilities for the remaining intestine.

It is evident that the intestine adapts to different physiological and anatomical changes [53,54,57]. However, patients may also have different renal clearances [58,59,60] and volumes of distribution [61] before and after surgery. Moreover, reports show that weight and obesity influence intestinal permeability and renal clearance [62,63,64]. The patients lost weight after RYGB, resulting in altered pharmacokinetic parameters [65].

In our model, we included the permeabilities for different intestinal segments. In the compound tab of GastroPlus^TM^ the effective permeability (*P_eff_*) can be calculated by the program using the drug structure or can be entered based on the literature. GastroPlus^TM^ is able to calculate human *P_eff_* from *P_app_* values for Caco-2 cells. By considering the intestinal absorption window in which the paracellular permeability can be included, it seems that the *P_eff_* accounts for only (or preferably) the jejunal segment of the intestine. This can be seen in Figure 7, where GastroPlus^TM^ clearly underestimates the observed plasma concentration–time profile, since the duodenum and jejunum are omitted. The inclusion of the measured permeabilities for every intestinal segment delivers a very good prediction in the healthy, the control, and also the post-RYGB groups. In the case of metformin, GastroPlus^TM^ can make a close prediction only using these drug-specific segmental-dependent permeabilities without having to make further adjustments to the model. It seems that the program is able to adapt to the new physiology with just the known values, without changing the pore size, pore density, or the absorptive surface area [34]. Since it is unknown to what extent these adaptations occur, it is difficult and non-ideal to change these values. Moreover, only some drugs [66,67,68] may show higher exposure after bariatric surgery, as metformin does [69], while for others, oral absorption may be impaired after surgery [70,71,72].

It seems that the absorption of drugs after surgery depends on several parameters. The weight but also the disease burden have an impact on intestinal permeability and clearance. The gut seems to adapt to the new physiology by increasing the epithelial surface area [54]. In the case of metformin, this might be an important factor. While the increased gastric pH may have an impact on other drugs, this is unlikely to be the case for metformin. Metformin is a strong base, which is protonated at a physiological pH value. It has a tendency to stick to the negatively charged intestinal epithelium, thus affecting drug absorption [12]. It was also reported that high concentrations are retained for several hours in the upper parts of the GI tract, showing depot-like behavior [14]. Furthermore, metformin is predominantly transported via the paracellular route (90%) [33]. If the rest of the GI tract is able to compensate for the missing segments, this may explain the observed plasma concentration–time profiles. Metformin will stick to the intestinal wall and be constantly absorbed via the paracellular route along the remaining small intestine. If needed, this could be simulated by increasing the transit time in different segments. Metformin is a substrate for various organic cation transporters and other influx transporters, whose activity may be enhanced under post-bariatric anatomical and physiological conditions, including an increase in pH [73].

If the permeabilities for every intestinal segment are known, they can be included in the gut physiology tab in GastroPlus^TM^. This can significantly improve the prediction of observed plasma concentrations. When the segmental-dependent permeabilities, the clearance, and other pharmacokinetic as well as physicochemical parameters of a certain drug are measured before and after surgery, they can be of great help in predicting and explaining the outcome. As we have shown here, implementing experimental in vivo data allows for accurate in silico predictions that do not rely on assumptions regarding the extent of gastrointestinal adaptations. Since each drug behaves differently in the GI tract, it is important to investigate drugs belonging to different biopharmaceutical classification system (BCS) classes to strengthen the prediction power of drug absorption post-surgery.

In conclusion, available simulation programs, such as GastroPlus^TM^, are potentially good tools to simulate and predict the absorption of different drugs even after bariatric surgery. They may allow for an appropriate and more optimal treatment for post-RYGB patients.

## Figures and Tables

**Table 1:** Input parameters before and after Roux-en-Y gastric bypass (RYGB) used in the compound window of GastroPlus^TM^. Control subject, individual with obesity; Log D, the distribution coefficient; and *P_app_*, apparent permeability.

Parameter	Input	Source
Dose (healthy)	500	[35]
Dose (control/RYGB subject)	1000	[10,34]
Dosage form	Immediate release	[10]
Molecular weight	129.17	ADMET Predictor 8.1
Log D (at a pH of 4.0)	−3.37	[32]
pKa	11.5	ADMET Predictor 8.1
Solubility	100.05 @pH 12.24	ADMET Predictor 8.1
Permeability	*P_app_* = 0.05 × 10^−5^ cm/s	[16,33]
Renal clearance (healthy)	0.5177 L/h/kg	[10,35,36,37,38,39,40]
Renal clearance (control)	0.18 L/h/kg	[10]
Renal clearance (RYGB)	0.258 L/h/kg	[10]
Body weight (healthy)	63.4 kg	[35]
Body weight (pre-RYGB)	114.6 kg	[10]
Body weight (post-RYGB)	104.0 kg	[10]
Volume of distribution (healthy)	1.784 L/kg	[41]
Volume of distribution (control)	1.0 L/kg	[10]
Volume of distribution (RYGB)	1.4 L/kg	[10]

**Table 2:** Parameters of the post-RYGB physiology. Changes from default are marked red.

Compartment	pH	Transit Time (h)	Volume (mL)	Length (cm)
Stomach	1.3 → 6.4	0.25 → 0.12	50.00 → 30	30 →18
Duodenum	6.00	0.26 → 0	48.25 → 0	15.00 → 0
Jejunum 1	6.20	0.95 → 0	175.3 → 0	62.00 → 0
Jejunum 2	6.40	0.73	139.90	62.00
Ileum 1	6.60	0.59	108.5	62.00
Ileum 2	6.90	0.43	79.48	62.00
Ileum 3	7.40	0.31	56.29	62.00
Caecum	6.40	4.50	52.92	13.75
Ascending colon	6.80	13.50	56.98	29.02

**Table 3:** The observed [10] and predicted pharmacokinetic parameters for the control and the adjusted post-RYGB group after a 1000 mg dose of metformin. Cmax, peak plasma concentration; Tmax, time to Cmax; and AUC, area under the curve.

Condition	Parameters	Observed Mean	Predicted Mean
Control	C_max_ (ng/mL)	1800	1598.2
	T_max_ (h)	3.0	2.4
	AUC_0–∞_ (ng/h/mL)	11,400	13,050
	AUC_0–24_ (ng/h/mL)	11,100	12,810
	Bioavailability (%)	27.80	27
Post RYGB	C_max_ (ng/mL)	2000	1781.5
	T_max_ (h)	3.0	2.7
	AUC_0–∞_ (ng/h/mL)	13,700	15,100
	AUC_0–24_ (ng/h/mL)	13,400	14,830
	Bioavailability (%)	41.80	40.60

## Data Availability

All the relevant data is included in the manuscript.
